# Epidemiology of Esophageal Cancer in Japan and China

**DOI:** 10.2188/jea.JE20120162

**Published:** 2013-07-05

**Authors:** Yingsong Lin, Yukari Totsuka, Yutong He, Shogo Kikuchi, Youlin Qiao, Junko Ueda, Wenqiang Wei, Manami Inoue, Hideo Tanaka

**Affiliations:** 1 Department of Public Health, Aichi Medical University School of Medicine, Nagakute, Aichi, Japan; 1 愛知医科大学医学部公衆衛生学; 2 Division of Cancer Development System, National Cancer Center Research Institute, Tokyo, Japan; 2 国立がん研究センター、発がんシステム研究分野; 3 The Fourth Affiliated Hospital, Hebei Medical University, Hebei Cancer Institute, Shijiazhuang, China; 3 河北医科大学第四医院・河北腫瘤研究所; 4 Department of Cancer Epidemiology, Cancer Institute/Hospital, Chinese Academy of Medical Sciences, Beijing, China; 4 中国医学科学院腫瘤医院; 5 Epidemiology and Prevention Division, Research Center for Cancer Prevention and Screening, National Cancer Center Research Institute, Tokyo, Japan; 5 国立がん研究センター、がん予防・検診研究センター; 6 AXA Department of Health and Human Security, Graduate School of Medicine, The University of Tokyo, Tokyo, Japan; 6 東京大学大学院医学系研究科、健康と人間の安全保障(AXA)寄附講座; 7 Division of Epidemiology and Prevention, Aichi Cancer Center Research Institute, Nagoya, Japan; 7 愛知県がんセンター研究所、疫学予防部

**Keywords:** esophageal cancer, epidemiology, risk factor

## Abstract

In preparation for a collaborative multidisciplinary study of the pathogenesis of esophageal cancer, the authors reviewed the published literature to identify similarities and differences between Japan and China in esophageal cancer epidemiology. Esophageal squamous cell carcinoma (ESCC) is the predominant histologic type, while the incidence of esophageal adenocarcinoma remains extremely low in both countries. Numerous epidemiologic studies in both countries show that alcohol consumption and cigarette smoking are contributing risk factors for ESCC. There are differences, however, in many aspects of esophageal cancer between Japan and China, including cancer burden, patterns of incidence and mortality, sex ratio of mortality, risk factor profiles, and genetic variants. Overall incidence and mortality rates are higher in China than in Japan, and variation in mortality and incidence patterns is greater in China than in Japan. During the study period (1987–2000), the decline in age-adjusted mortality rates was more apparent in China than in Japan. Risk factor profiles differed between high- and low-incidence areas within China, but not in Japan. The association of smoking and drinking with ESCC risk appears to be weaker in China than in Japan. Genome-wide association studies in China showed that variants in several chromosome regions conferred increased risk, but only genetic variants in alcohol-metabolizing genes were significantly associated with ESCC risk in Japan. A well-designed multidisciplinary epidemiologic study is needed to examine the role of diet and eating habits in ESCC risk.

## Abstract (ja)

食道がんの発生要因の解明を目的とした日中共同研究を実施するために、著者らは日本と中国で発表されている食道がん疫学文献をレビューした。組織型では日中両国とも扁平上皮がんが圧倒的に多く、腺がんの頻度が非常に低い。両国で行われた多くの研究によると、喫煙と飲酒が最も重要なリスク要因であることは一致している。しかし、がんによる負担、罹患率や死亡率、死亡の男女比、リスク要因、遺伝的感受性などの点において違いが認められる。全体に日本より中国の食道がん罹患率及び死亡率が高く、地域による罹患率及び死亡率の差も大きい。1987年から2000年までの年齢調整死亡率の低下は、日本より中国のほうが大きかった。中国では、リスク要因は多発地域と低発地域とでは異なるが、日本では地域によるリスク要因の違いが報告されていない。中国では飲酒と喫煙と食道がんとの関連が日本より強くない傾向がある。中国で実施されたゲノムワイド関連分析から幾つかの染色体のregionが感受性と関連すると報告しているのに対し、日本ではアルコール代謝関連遺伝子多型がリスクと有意に関連すると報告している。食事や食習慣の影響については学際的研究による検討が必要である。

## INTRODUCTION

Since the signing in November 2008 of a memorandum between the Ministry of Health (China) and the Ministry of Health, Labour and Welfare (Japan), the National Cancer Center (Tokyo) and Chinese Academy of Medical Sciences (Beijing) have each assembled a research group to facilitate collaboration on cancer epidemiology, prevention, and control. Areas of potential or ongoing collaboration include cancer registries, tobacco control, cancer epidemiologic studies and prevention, and environmental exposure assessment. After literature review and mutual field visits, the 2 research groups have reached an agreement on conducting a multidisciplinary study of the pathogenesis of esophageal and gastric cardia cancer in Hebei Province, China, a region that contains areas with some of the highest incidences of esophageal cancer in the world. To prepare for this collaborative work, the authors reviewed the published literature to identify similarities and differences in esophageal cancer epidemiology between Japan and China and generate hypotheses for further study.

Two major histologic types of esophageal cancer have been defined: esophageal squamous cell carcinoma (ESCC) and esophageal adenocarcinoma (EAC).^1^ While EAC has emerged as the major type in some Western countries, in Asia ESCC is the predominant type and EAC remains rare.^2^^–^^5^ ESCC and EAC share biologic features and some common risk factors such as cigarette smoking; however, they differ in geographic and demographic characteristics, risk factors, and pathogenesis.^6^ Because of the rarity of EAC, ESCC has been the subject of most studies in Japan and China. Although numerous studies have been conducted in both countries, very few have comprehensively compared the characteristics of esophageal cancer. We believe that such a comparison would better address unresolved questions in the field and provide new ideas for further studies.

In this review article, the term “esophageal cancer” is used to refer to ESCC unless EAC is specified. We compare the burden of esophageal cancer between Japan and China in terms of patterns of incidence and mortality, address factors associated with ESCC risk, based on epidemiologic studies conducted in each country, discuss prevention strategies, and propose 3 avenues for future studies of esophageal cancer pathogenesis.

## ESOPHAGEAL CANCER: INCIDENCE, MORTALITY, AND TRENDS

**Figure 1.**
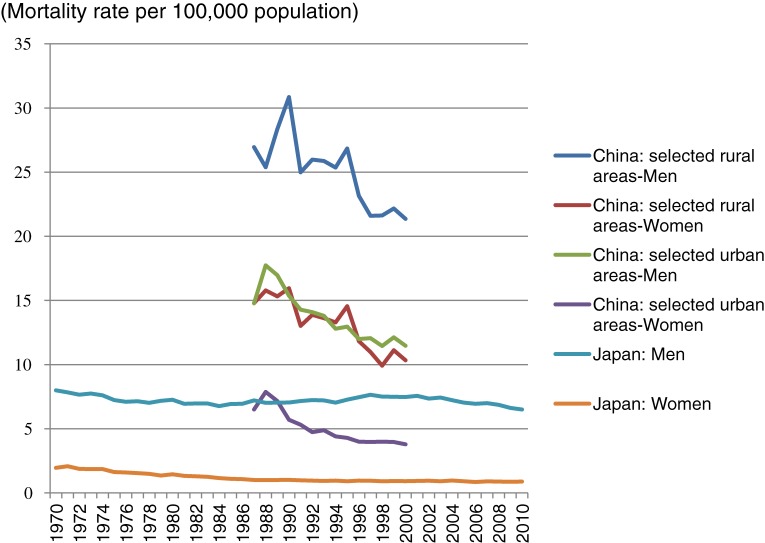
Age-standardized mortality rates in Japan and China. Source: World Health Organization mortality database.

Overall incidence and mortality rates for esophageal cancer are higher in China than in Japan. According to the Globocan,^7^ esophageal cancer is the tenth most common malignancy and the seventh most common cause of cancer death in Japan, with an estimated 17 497 new cases and 11 746 deaths in 2008. The estimated overall age-adjusted incidence rate (standardized for world population) in 2008 was 5.7 per 100 000 population. Data provided by the Center for Cancer Control and Information Services show that the age-adjusted incidence rate (per 100 000 population) increased from 8.3 to 11.7 during the period 1975–2006 among Japanese men but changed little among Japanese women, who had an estimated rate of approximately 1.5 during that period.^8^ During the period 1950–2010, the annual number of deaths continued to increase among Japanese men but did not significantly change among Japanese women (data not shown; available from the World Health Organization [WHO] mortality database). The male-female ratio of esophageal cancer mortality is approximately 6:1. However, according to the WHO mortality database,^7^ during the period 1970–2010, there was a gradual decrease in age-adjusted mortality among men after 1996 and a gradual decrease among women throughout the entire period (Figure 1).

In contrast, esophageal cancer is the fourth most frequently diagnosed cancer and the fourth leading cause of cancer death in China, with an estimated 259 235 new cases and 211 084 deaths in 2008. The estimated age-adjusted incidence rate in 2008 was 16.7 per 100 000 population. As compared with Japan, crude and age-standardized incidence rates for esophageal cancer markedly vary by geographic variation in China (Table 1). Throughout China, incidence rates are generally higher in rural areas than in urban areas. In particular, certain rural areas in Henan, Hebei, and Shanxi in Central North China have among the highest incidence rates in the world (>100 per 100 000 population). For example, Cixian has an incidence rate 18 times that of Beijing or Shanghai (Table 1). Previous mortality studies showed that areas with age-adjusted rates greater than 30 per 100 000 population were distributed in Sichuan, Anhui, Jiangsu, Hubei, Fujian, Guangdong, and Xinjiang provinces.^9^ In the present study, we use this rate (>30 per 100 000 population) as the definition of a “high incidence area”.

**Table 1.** Crude and age-standardized incidence rates of esophageal cancer in Japanese and Chinese populations

Area	Period	Men					Women				
	
		Crude	ASR	MV (%)	DCO (%)	MI (%)	Crude	ASR	MV (%)	DCO (%)	MI (%)
**Japan**											
Aichi Prefecture	1998–2002	9.2	6.4	82.1	15.5	69.0	1.4	0.8	64.9	27.0	70.3
Fukui Prefecture	1998–2002	12.2	6.0	89.4	3.7	56.9	2.9	1.1	82.0	9.8	55.7
Hiroshima	1996–2000	18.4	12.1	95.4	1.4	56.9	3.7	2.0	92.5	1.9	53.8
Miyagi Prefecture	1998–2002	28.6	15.4	85.2	8.4	57.7	5.4	2.2	79.5	12.8	56.6
Nagasaki	1998–2002	21.7	10.7	90.3	4.8	66.0	3.1	1.2	85.4	7.3	68.3
Osaka Prefecture	1998–2002	19.0	10.8	76.3	12.0	76.7	3.7	1.7	71.1	16.8	72.9
Saga	1993–1997	14.7	8.2	85.0	9.0	51.0	2.1	0.9	84.0	11.0	56.0
Yamagata Prefecture	1998–2002	29.7	13.0	87.1	8.4	68.5	4.7	1.6	76.0	14.0	62.7

**China**											
Beijing	1993–1997	14.6	10.2	74.0	2.0	71.0	6.4	4.0	67.0	3.0	70.0
Changle^a^	1993–1997	21.0	30.1	55.0	—	87.0	8.2	8.9	47.0	—	87.0
Cixian^a^	1993–1997	133.9	183.8	75.0	3.0	72.0	105.0	123.1	70.0	6.0	72.0
Guangzhou	2000–2002	9.2	9.3	71.0	0.2	86.0	2.3	1.8	70.8	0.0	75.8
Qidong County	1993–1997	13.7	13.2	57.0	0.0	92.0	5.6	3.9	54.0	0.0	92.0
Shanghai	1998–2002	14.4	9.2	63.4	0.6	70.4	6.2	3.0	55.8	0.8	72.0
Zhongshan	1998–2002	16.0	16.5	95.5	0.0	—	1.8	1.9	95.0	0.0	—

Source: Cancer Incidence in Five Continents Vol. VIII and Vol. IX, IARC Scientific Publications No. 155 and No. 160.

ASR: age-standardized rate, per 100 000 population; MV: morphologic verification of diagnosis; DCO: death certificate only; MI: ratio of mortality to incidence registered.

^a^These 2 areas were defined as “high-incidence areas” (ASR >30 per 100 000 population).

**Figure 2.**
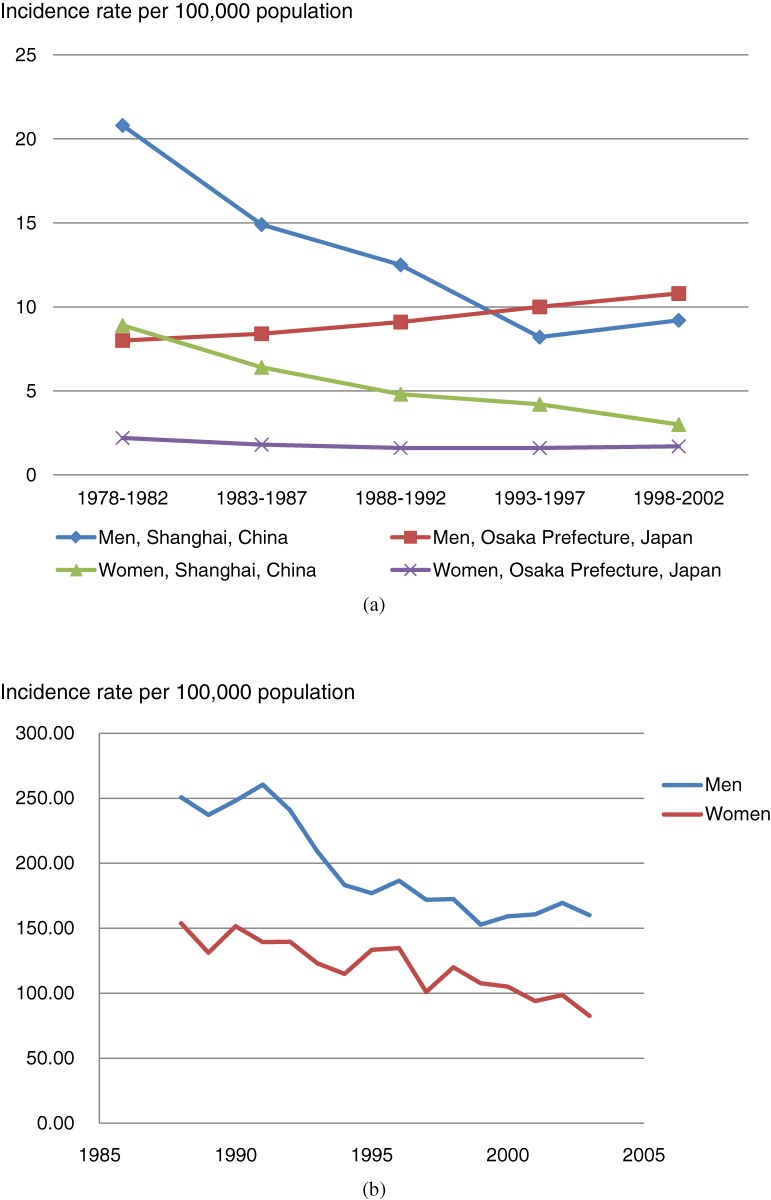
(a) Trend in age-adjusted incidence rates of esophageal cancer in Shanghai, China (representative low incidence area) and Osaka, Japan. Source: Cancer Incidence in Five Continents. (b) Trends in age-adjusted incidence rates of esophageal cancer in Cixian, a high-risk rural area in China. Source: He YT, et al. Trends in incidence of esophageal and gastric cardia cancer in high-risk areas in China. Eur J Cancer Prev. 2008;7:71–6. Reprinted with permission from the authors.

As compared with rural areas such as Cixian,^10^ cities like Shanghai and Beijing have experienced a greater decrease in esophageal cancer incidence over the past several decades. Using well-developed Shanghai cancer registry data, Zheng et al showed that the incidence of esophageal cancer had significantly decreased, by 59%, between 1975 and 1988.^11^ Notably, a comparison of cancer registry data from Osaka Prefecture, Japan and Shanghai showed that by 1998–2002, these areas had comparable incidence rates (Figure 2).

According to the WHO mortality database,^7^ age-adjusted mortality rates were higher in some rural areas than in urban areas. Both rural and urban areas showed a decline in mortality rates during the period 1987–2000 (Figure 1). The male-female ratio for esophageal cancer mortality in China is approximately 2:1.

## FACTORS ASSOCIATED WITH ESCC

### Squamous dysplasia

*Japan:* In Japan, controversy remains as to whether dysplasia should be recognized as a precursor lesion. Data are lacking on the prevalence of dysplasia in asymptomatic Japanese people, but a recent study of 1345 individuals who underwent a screening endoscopy during a health check-up found that 3% had dysplasia.^12^ The association between dysplasia and ESCC risk is unknown because no prospective data are available.

*China:* Cytologic and endoscopic screening in high-risk areas of China showed that it was possible to detect precursor lesions such as dysplasia in asymptomatic individuals with early-stage cancer.^13^ High-grade dysplasia is the principal precursor lesion and was shown to be closely associated with ESCC. In a prospective follow-up study in Linxian, China, squamous dysplasia was strongly associated with ESCC risk; the relative risk (RR) was 28.3 for individuals with severe dysplasia as compared with those with normal mucosa.^14^ Other studies estimated that SCC developed in 9% of patients with squamous dysplasia during a 15-year period^15^ and in 30% of patients with high-grade dysplasia during an 8-year period.^16^

### Alcohol consumption and cigarette smoking

*Japan:* The prevalence of current drinking was 36.4% among men and 6.9% among women, according to the 2009 National Health and Nutrition Survey.^17^ The prevalence of current smoking was 38.2% in men and 10.9% in women, according to the same survey. Alcohol consumption and cigarette smoking are 2 major risk factors for esophageal cancer in the Japanese population. A 2011 meta-analysis of 4 cohort studies and 8 case-control studies published between 1990 and 2010 showed that drinkers had a 3.3-fold increased risk of developing ESCC as compared with nondrinkers (Table 2).^18^ Moreover, all the included cohort studies reported a dose-response relationship between the amount of alcohol consumed, frequency of consumption, and ESCC risk.^18^

**Table 2.** Major risk factors for esophageal cancer in Japan and China

Risk factors	Information on strength of association
**Japan**	
Cigarette smoking	Summary RR for ever smokers was 3.01 (95% CI: 2.30–3.94), based on 4 cohort and 11 case-control studies [Ref 19]
Alcohol drinking	Summary RR for ever drinkers was 3.30 (95% CI: 2.30–4.74), based on 9 cohort and 9 case-control studies [Ref 18]
Gastric atrophy	Positive associations observed in 3 clinical studies [Refs 42–44], but no prospective cohort studies confirmed these associations.

**China**	
Low-incidence areas^a^	
Cigarette smoking	RR was 2.06 (95% CI: 1.11–3.82) for those who smoked for ≥40 years in a cohort of Shanghai residents [Ref 32]
Alcohol drinking	RR was 2.02 (95% CI: 1.31–3.12) for regular drinkers in a cohort of Shanghai residents [Ref 32]
Drinking tea at high temperature	OR was 3.1 (95% CI: 2.2–4.3) in a case-control study in Jiangsu Province [Ref 33], but definitive evidence is lacking
High-incidence areas^b,c^	
Cigarette smoking and alcohol drinking (probable modest association)	RR was 1.32 (95% CI: 1.15–1.51) for current smokers and 1.12 (0.83–1.51) for currents smokers of ≥20 cigarettes per day [Ref 29] No significant association among those who consumed alcohol in the previous 12 months [Ref 29]
Family history	RR was 1.42 (95% CI: 1.29–1.56) for individuals with family history of esophageal cancer [Ref 67]
Nutritional deficiency	High intake of meat, eggs, and fresh fruit associated with decreased risk [Ref 29]
Food mutagens including nitrosamine and its precursors	Ecologic studies showed that concentration of nitrate nitrogen was higher in high-incidence areas than in low-incidence areas [Refs 40, 41]

RR: relative risk; OR: odds ratio.

^a^In general, low-incidence areas are distributed in urban cities, including Beijing, Guangdong, Qidong county, Shanghai, and Zhongshan (Table 1).

^b^High-incidence areas are defined as areas with an age-standardized rate >30 per 100 000 population, including rural areas such as Cixian and Changle (Table 1).

^c^The main findings in high-incidence areas are based on a prospective study of risk factors for esophageal and gastric cancers in the Linxian General Population Trial Cohort in China [Ref ^29^].

A meta-analysis of 4 cohort studies and 11 case-control studies showed that the RR for current smokers relative to never smokers was 3.73 (95% CI, 2.16–6.43).^19^ A dose-response relationship was apparent in all 4 cohort studies and in 5 case-control studies. The synergistic effect of alcohol consumption and cigarette smoking on esophageal cancer risk is well documented in Japanese studies: a greater than 10-fold increased risk was observed among Japanese with both habits.^20^ The fraction of esophageal cancer associated with alcohol consumption and cigarette smoking was estimated at 84.8% among Japanese men and 51.6% among Japanese women.^21^ After reviewing all epidemiologic evidence, the Research Group for the Development and Evaluation of Cancer Prevention Strategies concluded that there was convincing evidence that alcohol consumption and cigarette smoking strongly increase the risk of ESCC in the Japanese population.^18^^,^^19^

Alcohol and its metabolic pathway have a central role in predisposing individuals to ESCC. Acetaldehyde, the primary metabolite of ethanol, forms adducts with DNA and is thus responsible for the carcinogenic effect of alcohol beverages.^22^ Polymorphisms in the genes that encode alcohol dehydrogenase (ADH) and acetaldehyde dehydrogenase (ALDH)—2 important enzymes in the alcohol-metabolizing pathway—may contribute to variation in the amount of acetaldehyde produced. Differences in the activity of these enzymes, and the potential of acetaldehyde to cause mutations, may explain why ESCC risk varies among individuals with the same level of alcohol consumption. Yokoyama and colleagues from Japan clearly showed that drinkers who were ALDH*1/2 heterozygotes had a significantly increased risk of developing ESCC.^23^

Since 2009, there have been 2 genome-wide association (GWA) studies reporting functional variants that were significantly associated with susceptibility to esophageal cancer in the Japanese population.^24^^,^^25^ The first GWA study identified 4q21-23 and 12q24 as susceptibility loci, in which 2 functional variants in *ADH1B* and *ALDH2* showed significant associations with ESCC risk (Table 3).^24^ *ADH1B* and *ALDH2* are crucial in the metabolism of alcohol. That study also found a strong gene–environment interaction: individuals who had genetic risk variants and were both smokers and drinkers had more than 100-fold the risk of developing ESCC. The second GWA study reported similar findings, ie, clear synergistic effects of *ADH1B* and *ALDH2* SNPs, alcohol consumption, and cigarette smoking on ESCC risk.^25^

**Table 3.** Findings from genome-wide association studies of esophageal cancer in Japan and China

References	Sample size	Ethnic group	Loci associated with susceptibility to ESCC
Wang et al 2004^56^	1077 ESCC cases 1733 controls	Chinese	*PLCE1* (10q23 rs2274223; per allele OR = 1.43 [1.37–1.49]) and *C20orf54* (20p13; per allele OR = 0.86 [0.82–0.90])
Abnet et al 2010^57^	2115 ESCC cases 3302 controls	Chinese	*PLCE1* (10q23 rs2274223; per allele OR = 1.34 [1.22–1.48])
Wu et al 2011^58^	2031 ESCC cases 2044 controls	Chinese	Identified 7 susceptibility loci on chromosomes 5q11 (rs10052657; OR = 0.67 for minor variant allele), 21q22 (rs2014300; OR = 0.70 for minor variant allele), 6p21 (rs10484761), 10q23 (rs2274223), and 12q24 (rs2074356, rs11066280) (OR = 1.30–1.56 for minor variant alleles)
Cui et al 2009^24^	1070 ESCC cases 2836 controls	Japanese	*ALDH2* (4q21-23, rs671; per allele OR = 1.67 [1.58–1.76]) and *ADH1B* (12q24, rs1229984; per allele OR = 1.79 [1.69–1.88])
Tanaka et al 2010^25^	1071 ESCC cases 2762 controls	Japanese	*ALDH2* (4q23, rs671; per allele OR = 1.78 [1.60–1.98]) and *ADH1B* (12q24.11–13, rs1229984; per allele OR = 1.82 [1.63–2.03])

ESCC: esophageal squamous cell carcinoma; GCA: gastric cardia cancer; OR: odds ratio; ALDH2: acetaldehyde dehydrogenase; ADH1B: alcohol dehydrogenase.

*China:* In low-risk urban areas like Shanghai, 1.9% of 74 942 women were current alcohol drinkers at baseline in the Shanghai Women’s Health Study.^26^ In another sample of 3953 Shanghai adults, 26.6% of men and 1.8% of women were current drinkers.^27^ Surveys of men in 2 rural areas showed prevalences of 61.4% and 68.2%, respectively.^28^ In Linxian, a representative high-incidence rural area in China, 23% of the 9584 baseline population reported drinking alcohol in the Linxian General Population Trial Cohort.^29^ Among women, 8% of esophageal cancer cases were current drinkers.^29^ According to the 2010 Global Adult Tobacco Survey (GATS), which included a nationally representative sample of individuals aged 15 years or older, the prevalence of current smoking was 56.1% among males and 2.2% among females in rural areas.^30^ In urban areas, prevalence was 49.2% among males and 2.6% among females.^30^ Overall, alcohol consumption and cigarette smoking have been shown to be associated with increased ESCC risk in the Chinese population. For alcohol consumption, the results from a meta-analysis of the association between alcohol consumption and cancer risks were published in 2011.^31^ For esophageal cancer, the meta-analysis included 34 case-control studies and 2 cohort studies. Most of the case-control studies found a positive association with alcohol consumption (summary odds ratio, 1.79; 95% CI, 1.47–2.17). However, the findings from the 2 cohort studies were inconsistent. In 1 cohort study, conducted in Linxian, no significant positive association was found between alcohol consumption and ESCC risk.^30^ By contrast, the other cohort study, conducted in Shanghai, reported a 2.02-fold risk of ESCC among current drinkers.^32^ In 2011, a case-control study of 1000 patients with ESCC and control subjects found that smoking and drinking were associated with a significantly increased risk of ESCC among men, but not among women, in Jiangsu Province, a high-risk area in China.^33^ The proportion of esophageal cancer attributed to alcohol drinking was estimated at 15.2% in Chinese men and 1.3% in Chinese women.^34^ For cigarette smoking, in a cohort study conducted in Linxian, the RR for ESCC was 1.34 (95% CI, 1.15–1.53) among ever smokers, and the risk increased with increasing years of cigarette smoking.^30^ The proportion of esophageal cancer attributed to cigarette smoking was estimated at 17.9% in Chinese men and 1.9% in Chinese women.^35^

### Diet and dietary habits

*Japan:* The association of diet and eating habits with ESCC risk in the Japanese population remains unclear. Among Japanese, high consumption of fruit and vegetables seems to protect against ESCC. The relationship of fruit and vegetable intake with ESCC was examined in the Japan Public Health Center-based Prospective Study, and the results showed that a 100-gram per day increase in consumption of total fruit and vegetables was associated with an 11% decrease in ESCC incidence.^36^ Intake of pickled vegetables, however, was not associated with ESCC risk in that study. Another prospective study found that consumption of green-yellow vegetables and fruit reduced the risk of esophageal cancer, but the association was not statistically significant.^37^

*China:* Nutritional deficiency is believed to have an important role in ESCC development, especially in high-risk areas. Studies in Linxian found that general malnutrition, as well as deficiencies in selenium, zinc, folate, riboflavin, and vitamins A, C, E, and B_12_, was associated with increased risk of ESCC.^38^ Since the 1970s, in areas of North Central China with exceptionally high incidence rates, efforts have focused on identifying food mutagens and environmental carcinogens. Earlier studies found high concentrations of nitrates and nitrites, the precursors of nitrosamine, in drinking water samples, and nitrosamine in food samples.^39^ Two ecologic studies, 1 conducted in Cixian^40^ and the other in Shexian,^41^ found that high concentrations of nitrate nitrogen in well water correlated with ESCC incidence. These findings highlighted the possible role of high levels of nitrate exposure in the pathogenesis of ESCC in high-risk areas.

### Other etiologic factors

*Japan:* Apart from alcohol consumption and cigarette smoking, drinking tea at a high temperature was associated with 1.6-fold increased risk of esophageal cancer in a cohort study.^42^ Three case-control studies consistently showed a strong, positive association between gastric atrophy and ESCC risk,^43^^–^^45^ but there have been no studies on the association between *Helicobacter pylori* and ESCC in the Japanese population. In previous studies that used a candidate-gene approach, genetic polymorphisms in alcohol-metabolizing genes, DNA repair genes, and folate-metabolizing genes were linked to ESCC risk.^46^^,^^47^

*China:* Epidemiologic studies of Chinese populations have examined the association of esophageal cancer with *H. pylori* infection,^48^ gastric atrophy,^49^ human papillomavirus infection,^50^ and drinking green tea at a high temperature^51^; however, the evidence is not sufficient to draw any definitive conclusions. A case-control study of Linxian residents showed no significant increased risk of ESCC among individuals infected with *H. pylori*.^48^ Previous studies yielded mixed results on the association between green tea consumption, consumption of hot drinks, and risk of esophageal cancer. While a cohort study conducted in Linxian did not find any significant association,^29^ a population-based case-control study in Jiangsu Province found that drinking tea at a high temperature significantly increased risk of esophageal cancer, after adjustment for confounding factors, including alcohol consumption and cigarette smoking.^51^

Numerous studies targeting certain genes have reported an association of genetic polymorphisms, including *CYP1A1*, *CYP2E1*, and *MTHFR*, with ESCC risk in the Chinese population.^52^ In particular, the ADH1*47 Arg, ALDH2*2, and *MTHFR* 677 TT genotypes appear to act synergistically with alcohol consumption.^53^^–^^55^

Since 2010, 3 GWA studies of esophageal cancer in the Chinese population have been published, and 10q23 was consistently identified as a susceptibility locus for ESCC.^56^^–^^58^ The main findings are summarized in Table 3. Variants at 10q23 in *PLCE1* were significantly associated with ESCC and gastric cardia cancer in GWA studies by Wang et al and Abnet et al.^56^^,^^57^ *PLCE1* encodes a phospholipase and is involved in regulating cell growth, differentiation, apoptosis, and angiogenesis. In addition to *PLEC1*, *C20orf54* on chromosome 20p13 was significantly associated with susceptibility to ESCC in the GWA study by Wang et al.^56^ The biologic function of *C20orf54* is not clear, but it may be involved in modulating riboflavin absorption. Furthermore, 3 susceptibility loci for ESCC—on chromosomes 5q11, 6p21, and 21q22—were recently identified in the GWA study by Wu et al in 2011.^58^

Epidemiologic studies in China suggest that gastric cardia adenocarcinoma (GCA) and ESCC have a similar geographic distribution in incidence and common risk factors.^29^^,^^59^^–^^61^ In particular, GCA was more prevalent in ESCC high-risk areas such as Linxian and Cixian.^10^^,^^29^ Case-control and cohort studies in high-risk areas reported that family history of esophageal cancer, low socioeconomic status, and low intake of vegetables and fruit were significant risk factors for GCA and ESCC.^29^^,^^61^ Interestingly, in the GWA studies by Wang et al and Abnet et al, variants in *PLCE1* were also significantly associated with GCA risk.^56^^,^^57^ These findings strongly suggest that the pathogenic processes of ESCC and GCA are similar.

## DISCUSSION

### Similarities and differences in esophageal cancer between Japan and China

International comparisons of cancer epidemiology are challenging. We closely examined patterns of incidence, mortality rates, and risk profiles to identify similarities and differences between Japan and China in esophageal cancer epidemiology. The identified similarities were as follows (Table 4). First, ESCC is the predominant histologic type, and the incidence of EAC is extremely low in both countries. Second, numerous epidemiologic studies in both countries have confirmed that alcohol consumption and cigarette smoking are important risk factors for ESCC. Third, although a number of putative risk factors have been investigated, such as gastric atrophy and drinking hot beverages, the associations have been inconsistent and inconclusive.

**Table 4.** Summary of similarities and differences between Japan and China in epidemiology of esophageal cancer

	Japan	China
**Similarities**		
Histologic type	ESCC: predominant histologic type	
Incidence and mortality: men vs women	Higher rates in men than in women	
Risk factors	Two established risk factors: cigarette smoking and alcohol drinking	

**Differences**		
Health burden	Relatively low vs other major cancers	High, especially in rural areas
Pattern of incidence/mortality according to geographic area	Not noted	Wide variations between rural and urban areas
Risk factor profiles according to geographic area	Not noted	Probably different
Strength of associations concerning major risk factors: cigarette smoking and alcohol drinking	Strong	Relatively weak, especially in high-incidence rural areas
Association with gastric cardia adenocarcinoma	Not noted	Reported in recent GWAS Studies
Loci associated with susceptibility to ESCC in GWAS	*PLCE1* and *C20orf54*	*ADH1B* and *ALDH2*
Prevention strategy	Smoking cessation and avoidance of excessive drinking, especially in individuals with certain susceptibility risk variants, such as ALDH 2*1 genotypes.	Diet, alcohol consumption, and cigarette smoking are essential components. In rural areas, must improve nutritional status, drinking water quality, food preservation, and cooking practices.

ESCC: esophageal squamous cell carcinoma; GWAS: genome-wide association study; PLCE1: phospholipase C epsilon 1; C20orf54: chromosome 20 open reading frame 54; ADH1B: alcohol dehydrogenase; ALDH2: acetaldehyde dehydrogenase.

Despite these similarities, there are obvious differences between Japan and China in many aspects of ESCC (Table 4). First, the health burden of esophageal cancer is greater in China than in Japan. Overall incidence and mortality rates are higher in China. Indeed, China alone accounts for about half of new cases worldwide and has many areas with incidence rates exceeding 100 per 100 000 population. In Japan, however, esophageal cancer appears to be less of a burden than other digestive malignancies, such as cancers of the liver, stomach, and colorectum. Among Japanese women, in particular, mortality from esophageal cancer is among the lowest of all cancers—even lower than that from leukemia. Second, variation in mortality and incidence patterns is greater in China than in Japan, eg, the decline in age-adjusted mortality rates was more apparent in rural areas in China than in Japan for the available period (1987–2000). Third, risk factor profiles may differ between high-incidence and low-incidence areas in China, although this is not the case in Japan. Overall, the association between smoking, drinking, and ESCC risk might be weaker in China than in Japan, where compelling evidence confirms the central roles of alcohol consumption and cigarette smoking. In China other potent, but unidentified, risk factors may exist and account for a considerable proportion of ESCC (especially in high-incidence areas), in light of the very low prevalences of smoking and drinking among Chinese women. Fourth, studies in high-risk areas of China have shown that gastric cardia cancer and ESCC have many similarities, including geographic distribution, environmental risk factors, and genetic susceptibility alleles. By contrast, no such findings have been reported in Japan. Fifth, GWA studies in China showed that variants in several chromosome regions confer increased risk, suggesting the involvement of multiple genes in the carcinogenic process. However, GWA studies in Japan found that ESCC risk was associated only with genetic variants in alcohol-metabolizing genes.

### Prevention strategies

Although screening for precursor lesions and detection of early-stage ESCC in selected populations is performed in both Japan and China, prevention remains the best way to decrease the burden of esophageal cancer. Epidemiologic evidence indicates that ESCC is preventable through risk factor modification. Given the above-mentioned differences in various aspects of ESCC, the components of a prevention strategy would be different in Japan and China. In Japan, the decisive roles of alcohol consumption and cigarette smoking in ESCC have been clearly demonstrated; thus, efforts should focus on smoking cessation and avoidance of excessive drinking, particularly among individuals who harbor certain susceptibility risk variants, such as ALDH 2*1 genotypes. Ideally, the strategy would evolve to personalized prevention based on different genetic backgrounds and varied sensitivity to environmental carcinogens. In China, improved diet and reduced alcohol consumption and cigarette smoking should constitute the essential components of a prevention strategy. Educating the general public regarding risk factor modification is urgently needed in rural areas. Although diet-related mechanisms are not fully understood, improving nutritional status and eating habits would reduce risk. Because nitrosamine, heterocyclic amines, and polycyclic aromatic hydrocarbons are known food mutagens,^62^ improving drinking water quality, food preservation, and cooking practices are also important strategies in high-incidence areas.

### Future research directions

Given the complex pathogenesis of esophageal cancer, we would like to highlight 3 important research areas for future studies. First, recent GWA studies of esophageal cancer in Japanese and Chinese populations have yielded novel insights into the pathogenesis of ESCC. While GWA studies in the Japanese population found that the major susceptibility variants are located in alcohol-metabolizing genes, GWA studies in the Chinese population did not replicate this finding. Instead, susceptibility to esophageal cancer may be determined by many variants in different genes that have mostly small effects. Differences in study methodology and the frequency or effect size of the alleles at a given locus may explain differences in findings from GWA studies in these countries. Extremely high incidence rates in certain areas of China suggest that high-risk variants remain to be discovered. With the increasing availability of next-generation sequencing technologies, it would be interesting to attempt to identify such high-risk variants. Furthermore, the functional significance of variants identified in extant GWA studies and their interaction with environmental exposures should be clarified in future studies.

Second, there is a great need for a multidisciplinary approach to address the complex role of diet and eating habits in esophageal cancer development. As compared with Japan and low-risk areas of China, a variety of different factors may contribute to development of esophageal cancer in high-risk areas of China. If smoking and drinking do indeed have minor roles, then a high prevalence of potent, but unidentified, factors might be contributing to the lingering high incidence in those areas. It is highly likely that nitrosamine and its precursors are very strong risk factors.^39^ Although evidence from ecologic studies suggests a correlation between nitrosamine precursors and ESCC incidence,^40^^,^^41^ very few studies have measured nitrosamine and its precursors and evaluated their associations with esophageal cancer risk. The main challenge for such studies is correctly determining exposure to nitrosamine from various sources, including exogenous exposure and endogenous formation. Endogenous formation of nitrosamine depends on a variety of factors, including nitrate and nitrite sources, oral bacteria activity, vitamin C level, and secondary amine.^63^^,^^64^ Moreover, the interaction between these factors remains largely unknown. To unravel the complex mechanisms underlying the nitrosamine–esophageal cancer association, we need to target the whole process by using improved epidemiologic methods, specific biomarkers, and biological pathway analyses. For example, DNA adductome analyses, combined with epidemiologic data on environmental exposure and lifestyle, would provide valuable information on exposure to exogenous or endogenous carcinogens such as nitrosamine.^65^

Third, while there is convincing evidence that *H. pylori* is strongly associated with increased risk of noncardia gastric cancer, studies of its association with ESCC have been limited and have yielded inconsistent results. One mechanism to explain the association between *H. pylori* infection and the increased risk of gastric cancer is that hypochlorhydria in individuals with long-term *H. pylori* infection allows overgrowth of other bacteria, which increasingly convert ingested nitrites to N-nitrosamines.^66^ Determining whether this mechanism can also be applied to ESCC warrants further study. Moreover, prospective studies are needed to address the role of *H. pylori* infection and gastric atrophy in ESCC development.

In summary, while evidence from the latest GWA studies has advanced our understanding of esophageal cancer pathogenesis, the best strategy for preventing esophageal cancer in Japan and China remains risk factor modification, namely smoking cessation and avoidance of excessive drinking. It is hoped that the role of diet and eating habits will be clarified in a future well-designed multidisciplinary epidemiologic study.

## ONLINE ONLY MATERIALS

Abstract in Japanese.
