# Supplementary material for: Epidemiology of Esophageal Cancer in Japan and China
Source: J Epidemiol. 2013 Jul 5;23(4):233–42. doi: 10.2188/jea.JE20120162 (PMC3709543; doi:10.2188/jea.JE20120162)
Supplement: Abstract in Japanese. [file je-23-233-s001.pdf]

日本と中国における食道がんの疫学

林櫻松[1]、戸塚ゆか里[2]、贺宇彤[3]、菊地正悟[1]、乔有林[4]、上田純子[1]、 魏文强[4]、井上真奈美[5,6]、田中英夫[7]

[1]愛知医科大学医学部公衆衛生学
[2] 国立がん研究センター、発がんシステム研究分野
[3]河北医科大学第四医院・河北腫瘤研究所
[4]中国医学科学院腫瘤医院
[5]国立がん研究センター、がん予防・検診研究センター
[6]東京大学大学院医学系研究科、健康と人間の安全保障(AXA)寄附講座
[7]愛知県がんセンター研究所、疫学予防部

食道がんの発生要因の解明を目的とした日中共同研究を実施するために、著者らは日本と中国で発表されている食道がん疫学文献をレビューした。組織型では日中両国とも扁平上皮がんが圧倒的に多く、腺がんの頻度が非常に低い。両国で行われた多くの研究によると、喫煙と飲酒が最も重要なリスク要因であることは一致している。しかし、がんによる負担、罹患率や死亡率、死亡の男女比、リスク要因、遺伝的感受性などの点において違いが認められる。全体に日本より中国の食道がん罹患率及び死亡率が高く、地域による罹患率及び死亡率の差も大きい。1987 年から 2000 年までの年齢調整死亡率の低下は、日本より中国のほうが大きかった。中国では、リスク要因は多発地域と低発地域とでは異なるが、日本では地域によるリスク要因の違いが報告されていない。中国では飲酒と喫煙と食道がんとの関連が日本より強くない傾向がある。中国で実施されたゲノムワイド関連分析から幾つかの染色体の region が感受性と関連すると報告しているのに対し、日本ではアルコール代謝関連遺伝子多型がリスクと有意に関連すると報告している。食事や食習慣の影響については学際的研究による検討が必要である。

キーワード：食道がん、疫学、危険因子
